# The effect of peer education based on adolescent health education on the resilience of children and adolescents: A cluster randomized controlled trial

**DOI:** 10.1371/journal.pone.0263012

**Published:** 2022-02-02

**Authors:** Yinshuang Tang, Hua Diao, Feng Jin, Yang Pu, Hong Wang

**Affiliations:** School of Public Health and Management, Chongqing Medical University, Research Center for Medicine and Social Development, Collaborative Innovation Center of Social Risks Governance in Health, Chongqing Medical University, Chongqing, China; University of Southern Queensland, AUSTRALIA

## Abstract

**Background:**

An increasing number of children and adolescents have reported mental health problems, and resilience is a protective factor against these problems. Therefore, the aim of the study is to verify the effect of peer education based on adolescent health education on adolescent resilience.

**Method:**

A cluster randomized controlled trial was conducted including 1,613 students who were divided into an intervention group (19 classes, 732 participants) and a control group (24 classes, 881 participants). One-year peer education was performed in the intervention group, and the control group had no interventions. The Resilience Scale for Chinese Adolescents by Yueqin Hu and a self-designed basic information questionnaire were used to collect data. Chi-square test and rank-sum test were used to compare the differences of demographic characteristics between the two groups. A linear mixed model was used to compare the changes of resilience between the two groups after intervention, and the intra-cluster correlation coefficient (ICC) was calculated. A generalized linear mixed model (GLMM) was used to verify the effect of peer education on adolescent resilience. The significance was set at *P* < 0.05.

**Results:**

After intervention, compared with the control group, the intervention group showed significant improvement in target focus, emotion adjustment, interpersonal assistance and total resilience (*P* < 0.05). The ICC range was 0.003 to 0.034. The GLMM results indicated that peer education based on adolescent health education had significant effects on adolescents’ target focus (*β* = 0.893, *P* = 0.002), emotional adjustment (*β* = 1.766, *P* < 0.001), interpersonal assistance (*β* = 1.722, *P* = 0.016) and total mental resilience (*β* = 5.391, *P* < 0.001), and the effect was greater for boys than for girls.

**Conclusions:**

Peer education based on adolescent health education is effective for improving adolescents’ target focus, emotional adjustment, interpersonal assistance, and total resilience, especially for males. Future research should devote more attention to positive cognition and family support as well as gender differences.

## Introduction

In China, with the rapid development of social economy and the change of social structure in recent decades, social pressure and competition have gradually increased. For adolescents, higher academic requirements and psychological pressure lead to the increase of negative emotions, and mental health problems are becoming increasingly prominent [1, 2]. It is estimated that more than 100 million of China’s 1.3 billion people suffer from mental disorders, of which about 16 million are seriously ill [3]. Another national epidemiological study has found that 15% of children in China suffer from mental health problems, and the prevalence of anxiety and other diseases is rising [4].

With the development of positive psychology, researchers have devoted more attention to the positive effect of negative events on the mental health of adolescents [5, 6]. In other words, not all individuals in adversity will experience negative outcomes such as anxiety and depression, and some individuals will instead experience better positive outcomes as a result of negative circumstances [7]. Therefore, some experts have proposed the term “resilience”, which is defined as the ability to withstand and recover from adverse environments in an effective manner [8]. As one of the protective factors of mental health [9], it can protect individuals from negative psychological problems such as stress and anxiety, and promote high self-esteem to reduce depressive symptoms [10, 11]. Previous studies have shown that resilience is influenced by a large number of influencing factors, including protective factors such as harmonious family relationships, friendly peer relationships, and positive coping skills [12, 13].

Adolescents are at a critical point in the transition from child to adults, involving a variety of physiological, psychological, and social function upheavals (e.g., genital development and pubic hair growth) that are stressful events for them [14, 15]. A robust body of research indicates that pubertal status is a key predictor of various internalizing and externalizing problems for adolescents that influence the resilience of adolescents [16, 17]. For instance, a study with 1,420 subjects, conducted by Angold et al. [18], showed that girls at Tanner 3 were over three times more likely to contact depressive disorders than girls in the earlier Tanner stages, independent of the age at which they entered stage 3. A growing body of literature implies that pubertal timing of adolescents occurs earlier [19, 20]. Earlier pubertal timing will increase the risk of internalizing and externalizing problems, aggravating the negative influence of adolescent changes on the mental health of adolescents [21–23]. For example, Mendle found that earlier pubertal timing had a significant association with depression and anxiety [24]. Therefore, we consider improving resilience by increasing positive adolescence-related knowledge, attitudes and behaviors.

The Healthy China Action (2019–2030) plan explicitly mentions initiatives to promote mental health and student health [25]. However, although the government has introduced important policies and paid more attention to mental health, more research and guidance are needed to build an interrelated and efficient mental health system [2]. A large number of previous studies have found that traditional health education in school classroom can effectively promote mental health such as resilience, anxiety and depression [12, 26, 27], and health risk behaviors such as smoking, alcohol abuse and unintentional injury [28–30], but the effect is incomplete and often short-term. Schools are key places to improve adolescent health, but classroom education is also challenging as schools increasingly focus on indicators of academic achievement [31]. After puberty, adolescents spend more time with their peers and have a stronger sense of identity with their peers [32], which suggests that peer education may be an effective way to improve the mental health of Chinese adolescents.

Peer education is defined as “sharing experiences and learning among those of a similar age, living environment, and culture with something” [33], which is based on social cognitive theory [34] that shows that the interactions and observations of others can impact the behavior and attitude of individuals. A large number of studies have shown that peer education is extremely effective in some domains, such as prevention of chronic diseases and dissemination of sexual knowledge [33, 35, 36]. This suggests that it is of great practical significance to adopt the method of peer education to intervene the resilience of Chinese adolescents. In most previous clinical trials, participants were randomized as individuals to receive different interventions. However, it is worth noting that sometimes individual assignment is not possible or desirable. Indeed, many educational evaluations are conducted in naturally occurring clusters (such as classes or schools), and the only practical way to conduct a randomized controlled trial (RCT) is to use cluster assignment. Cluster randomization is often used to avoid contamination between those who receive intervention and those who do not [37]. Using clusters rather than individuals as random units has proven to be a more efficient and economical option [38].

In summary, the main aim of this study was to conduct a cluster randomized controlled trial (cluster RCT) to verify the intervention effect of peer education based on adolescence-related knowledge, attitudes, and behaviors on adolescents’ psychological resilience. The main outcomes were at the individual and cluster levels of adolescents.

## Methods

### Sample size

For superiority trials comparing the means of the two groups, the following formula was used to calculate the sample size [39, 40]:

$$n={\left[\frac{(U_{1-\alpha }+U_{1-\beta })\sigma }{\delta }\right]}^{2}\times \frac{1+C}{C}$$ (1)

In formula (1), σ is defined as standard deviation; δ is referred to as a clinically meaningful low or high limit that is the width of the 95% confidence interval equally; c is the ratio of sample cases between intervention group and control group that is equal to 1 in this study. In our research, α and β were 0.05 and 0.10, respectively, so U_1-α_ and U_1-β_ were 1.64 and 1.28, respectively. Pre-surveys revealed that σ and δ are 14.85 and 3.14, respectively. Therefore, a sample size of at least 275 was calculated for both groups.


DE=1+(m−1)ρ (2)

Considering that this study was a cluster RCT, the sample size should be increased through design effect (DE) to ensure the efficiency of the test [41, 42]. When the average cluster size (m) is 40 and the intra-cluster correlation coefficient (ICC) is 0.05, the calculated DE is 2.95. The DE was multiplied by the sample size without clustering effect (n), resulting in a sample size of 811 students in each group and a cluster number of 20 in each group.

### Study design, participants, and processes

A cluster RCT was conducted using 4 schools (2 primary schools, 2 middle schools) in the Qijiang district of Chongqing. The inclusion criteria were four comparable schools in geographical distribution, educational philosophy and cultural level. Stratified by primary and junior high schools, 4 schools were randomly divided into the intervention group with peer education intervention and the control group without any intervention (blank control group). Finally, the intervention group and the control group each included a primary school and a middle school. All participants in the intervention group received peer education intervention, and all participants in the control group received no intervention. The survey was expected to take a year and a half. Considering that students in grade 6 and grade 9 will be lost to follow up due to entering a different school, they were not included in this study. The inclusion criteria were students in grades 4–5 in primary school and grades 7–8 in middle school who were able to complete the questionnaire independently. A total of 1,678 students in selected schools were eligible to participate in this study, but 8 individuals were excluded due to intellectual disabilities and being illiterate (exclusion criteria). Therefore, 758 and 912 students were included in the intervention group and control group, respectively.

**Fig 1:**
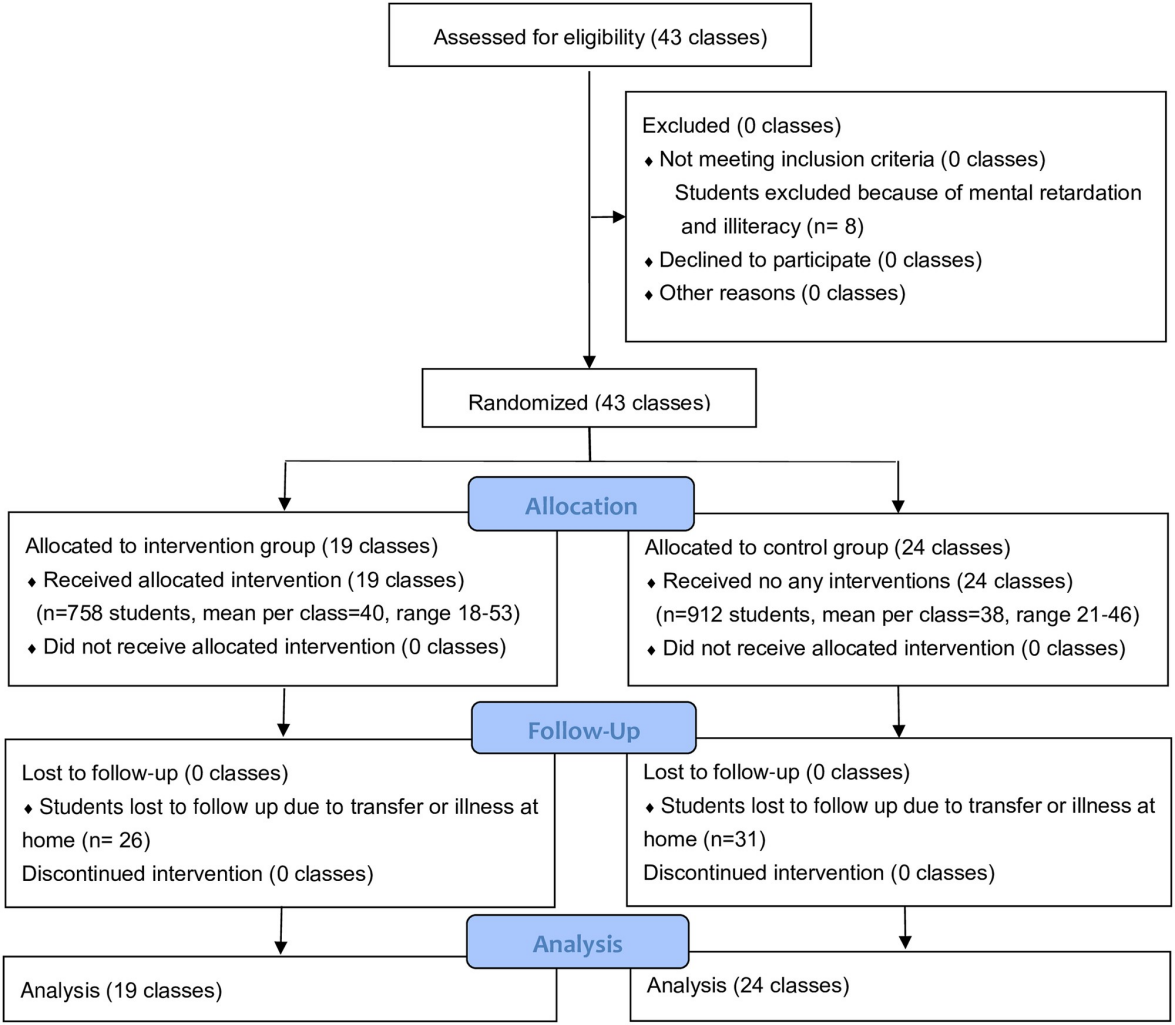
Flow chart of study.

At baseline in December 2017, with the help of school teachers, the researchers led 758 students (19 classes) in the intervention group and 912 students (24 classes) in the control group to complete the questionnaire, which mainly included the participants’ general demographic characteristics (gender, family economic status, parenting style, etc.) and psychological resilience related questions. Most participants completed the questionnaire within 40 minutes, with no obvious logical errors or missing items. In April 2,018, peer educator training was conducted by experts in the health of children and adolescents. The last follow-up was conducted in May 2019, one year after the intervention, 732 students (26 missings) in the intervention group and 881 students (31 missings) in the control group were investigated with the same questionnaire as the baseline. The reason for missing data was changing schools and homesickness. Finally, the data analysis included 1,613 students, as shown in Fig 1. The study was approved by the Biomedical Ethics Committee of Peking University (IRB 00001052–13,034) and the ethical committee of Chongqing Medical University, and written informed consent was obtained from students and their parents before investigation in the research. The trial was pre-registered in ClinicalTrials.gov before recruitment started (identifier NCT02343588).

### Peer education involving adolescent health education

In the intervention group schools, negotiating with head teachers in every class, 4 excellent, responsible and well-communicated students, including 2 boys and 2 girls, were selected as peer educators who served on the class committee, actively participated in extracurricular activities and had activity organization experiences. The training session for peer education activities was divided into primary school group and middle school group. The training teacher of each group was composed of 1 professor from Chongqing Medical University and 3 graduate students from the research group. The training forms were intensive teaching, group discussion and knowledge competition. The training content was divided into four parts: adolescent physical health knowledge, adolescent mental health knowledge, healthy behavior and lifestyle, and peer education knowledge and skills. Each training content lasted about 1 hour. The overall framework of the content and form of health intervention for primary and secondary school students was the same, but in the actual training and intervention process, different emphases were planned according to the physical and mental development characteristics of primary and secondary school students. For example, breast development, pubic hair growth and puberty growth spurt were taught to primary school students through games and role plays, while menarche, first spermatorrhea and sexual psychology were taught to junior middle school students through knowledge competition and group discussion [43–45]. At the end of the peer education training session, the training effect of 76 peer educators was evaluated through a real-time questionnaire survey. The results showed that the correct rate of adolescent physiological health knowledge such as body hair growth, menstruation, spermatorrhea and other major mental health problems such as anxiety, irritability, depression were significantly improved for both boys and girls. On the other hand, the attitude score of correctly facing adolescent problems has also been significantly improved (see S1 File). In addition, we also tested the peer educators’ learning of knowledge and skills related to peer education through interactive games, and the results showed that peer educators initially had the ability to carry out peer education activities. According to the theory of knowledge, attitudes, and practices (KAP) in behavior change models, knowledge is the foundation, attitude is the motivation and behavior is the goal. Knowledge improvement and attitude change contribute to the formation of individual behavior and skills [46]. It is suggested that the training of knowledge and attitude of adolescence and knowledge and skills of peer education activities can help peer educators to develop a healthy lifestyle and develop their ability to carry out peer education activities.

After the peer education training course, peer educators used the knowledge and skills learned in the training course and materials provided by the research group to carry out intervention activities for each class. Regarding the forms of peer education, a knowledge quiz game, group discussion, sitcom performance, self-designed poster exhibition and so on were used to conduct activities, with the provision of knowledge quiz software, group discussion cases and analysis results, role-playing scripts, and adolescence-related health education slides. Peer educators spent the class meeting or spare time conducting activities among their peers and were required to record key information of every activity, such as the number of participants, context of activity, and satisfaction and advice of participants. Importantly, the content and format we selected is unique and has never been carried out in the primary and secondary schools of the district and county. Additionally, educators had to perform at least 2 activities per month. During the intervention, peer education activities were tracked and documented by psychologists and research members through online interviews or field visits. Then, we conducted secondary training on October 2,018 to intensify relevant knowledge and teach them how to cope with stress and negative events. During the 1-year intervention, we performed supervision twice per semester to evaluate the process of peer education activities and to collect activity records.

With respect to the content of peer education sessions, based on the "Six Strategies Training Program" by Henderson and Milstein [47] and the key life period in which adolescents are transitioning from child to adult, physiological knowledge, psychological health education, and health lifestyles were included as the targeted intervention content. Physiological health education involves growth spurts, development of secondary sexual characteristics, acne treatment, treatment of breast development, menstruation, dysmenorrhea for girls, treatment of beard growth and seminal emission, and cleaning of the private parts. Psychological health education includes the process of psychological development during adolescence and the treatment of psychological problems such as tension, anxiety and conflicts with parents, teachers, and peers; a healthy lifestyle involves a balanced diet, reasonable exercise, and good sleep.

### Measurements

Psychological resilience was assessed through the Resilience Scale for Chinese Adolescents (RSCA) by Yueqin Hu [48] according to Chinese culture, which included 27 items and 5 factors (F1-F5). F1- Target focus, which means sticking to goals, making plans and focusing on problem-solving in difficult situations; F2—Emotional adjustment, which refers to the control and adjustment of emotional fluctuations and pessimism in difficult situations; F3—Positive cognition refers to a dialectical and optimistic attitude towards adversity; F4—Family support, which refers to the tolerance, respect and supportive attitude of family members; F5- Interpersonal assistance, which means that individuals can obtain help or vent their emotions through meaningful interpersonal relationships. All items were scored on a 5-point scale. The principles of assignment are as follows: positive entries have 5 options: totally inconsistent (1 point), comparatively inconsistent (2 points), unclear (3 points), comparatively consistent (4 points), and fully consistent (5 points). Some items were reverse scored: totally inconsistent (5 points), comparatively inconsistent (4 points), unclear (3 points), comparatively consistent (2 points), and fully consistent (1 point). The total score ranged from 27 to 135, with a higher score indicating a higher level of psychological resilience. We tested the reliability of this scale in our research sample, with the resultant Cronbach’s alpha of target focus, emotional control, positive cognition, family support, interpersonal assistance, and the overall scale being 0.765, 0.648, 0.709, 0.590, 0.666 and 0.818, respectively.

According to the influencing factors of resilience [49, 50], following sociodemographic characteristics were collected through self-designed questionnaires: age (continuous variable); participants’ education level (primary school/middle school); sex (male/female); whether the individual is an only child (Yes/No); relationship between parents and with parents (disharmonious/moderate/harmonious) linked to the number of conflicts; parents’ education level (junior high school or lower/senior high school and technical secondary school/college or higher); family economic status (good/moderate/bad); academic achievements (good/moderate/bad); the number of close friends (≤2/3-5/≥6); and parenting style (democratic/autocratic/doting/disregardful).

### Quality assurance

In the design of the study, experts in epidemiology, statistics, child and adolescent health, psychology and pedagogy were consulted extensively, and the plan was formulated in combination with the discussion of the research group. Before the investigation, all investigators received uniform standardized training to ensure the quality of the investigation. During the investigation, with the cooperation of the school leaders, the investigator explained the matters needing attention to fill in the questionnaire to the research subjects and guided the students to complete the questionnaire. The questionnaires were collected and checked by the members of the research group, and the questionnaires with obvious logical errors and missing items were returned to the students for revision in time. After the survey, all survey data were recorded and checked by two groups of investigators using EpiData 3.1 software. The subsequent processing of outliers and statistical analysis of the data were supervised by epidemiologists and statistical experts.

### Data analysis

EpiData 3.1 was used for data collection and collation, SAS 9.4 and SPSS24.0 were used for statistical analysis. The quantitative data were expressed by mean and standard deviation, and the qualitative data were expressed by number of cases and percentage. Propensity Score Matching (PSM) was used for data matching if baseline information differed significantly between the intervention and control groups. T test and one-way ANOVA were used for the difference between groups of continuous variables with normal distribution, and rank sum test was used for the difference between groups of continuous variables with non-normal distribution. Chi-square test or Fisher’s exact test was used for differences between groups of categorical variables. A linear mixed model was used to compare the change of resilience score between the two groups after intervention, and the cluster effect value ICC was calculated. After determining the changes between baseline and final follow-up, the effectiveness of peer education intervention on adolescent resilience was tested using a generalized linear mixed model (GLMM), with the change value of resilience score as the dependent variable and the intervention style and other unmatched inter-group imbalance factors as independent variables. The value of ɑ is equal to 0.05.

## Results

### Sociodemographic characteristics

The average age of the participants in this study was 12.49 ± 1.67 years. In the intervention group, the average number of activities per class was 14.81 (range: 10–29). Each class consisted of 4.38 posters on average, with minimum values of 2 posters and maximum values of 8 posters per class.

Table 1 shows the distribution of sociodemographic characteristics between the intervention group and the control group. Significant differences were found in participants’ age, “relationship between parents”, “relationship with father”, “father’s education level”, “parenting style”, and “family economic status” (*P* < 0.05). These unbalanced factors were controlled in the mixed model.

**Table 1:** The distribution of sociodemographic characteristics between intervention group and control group (n = 1,613).

Characteristics	Control (n = 881)	Intervention (n = 732)	*P*
**Educational degree**			
**Primary school**	345 (52.35)	314 (47.65)	0.130^a^
**Middle school**	536 (56.18)	418 (43.82)	
**Sex**			
**Male**	438 (53.68)	378 (46.32)	0.440^a^
**Female**	443 (55.58)	354 (44.42)	
**Only child**			
**Yes**	165 (57.49)	122 (42.51)	0.280^a^
**No**	716 (54.00)	610 (46.00)	
**Relationship between parents**			
**Harmonious**	773 (56.34)	599 (43.66)	0.002^a^
**Moderate**	86 (43.00)	114 (57.00)	
**Disharmonious**	22 (53.66)	19 (46.34)	
**Relationship with father**			
**Harmonious**	751 (56.51)	578 (43.49)	0.004^a^
**Moderate**	108 (45.96)	127 (54.04)	
**Disharmonious**	22 (44.90)	27 (55.10)	
**Relationship with mother**			
**Harmonious**	776 (55.07)	633 (44.93)	0.610^a^
**Moderate**	78 (50.98)	75 (49.02)	
**Disharmonious**	27 (52.94)	24 (47.06)	
**Father’s educational level**			
**Middle school or lower**	579 (58.02)	419 (41.98)	0.001^a^
**High/technical secondary school**	264 (48.00)	286 (52.00)	
**Junior college or higher**	38 (58.46)	27 (41.54)	
**Mother’s educational level**			
**Middle school or lower**	578 (56.12)	452 (43.88)	0.280^a^
**High/technical secondary school**	281 (51.94)	260 (48.06)	
**Junior college or higher**	22 (52.38)	20 (47.62)	
**Parenting style**			
**Democracy**	648 (57.09)	487 (42.91)	0.001^a^
**Autocratic**	132 (45.21)	160 (54.79)	
**Doting**	77 (58.78)	54 (41.22)	
**Disregardful**	24 (43.64)	31 (56.36)	
**Family economic status**			
**Good**	287 (50.98)	276 (49.02)	<0.001^a^
**Moderate**	434 (52.93)	386 (47.07)	
**Bad**	160 (69.57)	70 (30.43)	
**Academic achievements**			
**Good**	240 (53.93)	205 (46.07)	0.680^a^
**Moderate**	442 (54.10)	375 (45.90)	
**Bad**	199 (56.70)	152 (43.30)	
**Number of close friends**			
**≤2**	188 (52.22)	172 (47.78)	0.210^a^
**3–5**	388 (57.14)	291 (42.86)	
**≥6**	305 (53.14)	269 (46.86)	
**Age**	12.67±1.71	12.27±1.60	<0.001^b^

^a^ *P* values were calculated using the chi-square test.

^b^ *P* values were calculated using the rank sum test.

Quantitative data were expressed by “mean ± standard deviation”, while qualitative data were expressed by “n (%)”.

### Resilience changes in the intervention group and control group

In this study, the demographic characteristics of the intervention group and the control group were significantly different (Table 1), and the changes in resilience of the two groups could not be directly compared. Therefore, the PSM method was used to match the data of the two groups, and the demographic characteristics of the two groups were comparable after matching (Table 2).

**Table 2:** Distribution of sociodemographic characteristics between intervention and control groups after PSM (n = 1,338).

Characteristics	Control (n = 669)	Intervention (n = 669)	*P*
**Educational degree**			
**Primary school**	293 (50.34)	289 (49.66)	0.825^a^
**Middle school**	376 (49.74)	380 (50.26)	
**Sex**			
**Male**	342 (49.78)	345 (50.22)	0.870^a^
**Female**	327 (50.23)	324 (49.77)	
**Only child**			
**Yes**	121 (50.42)	119 (49.58)	0.887^a^
**No**	548 (49.91)	550 (50.09)	
**Relationship between parents**			
**Harmonious**	553 (49.91)	555 (50.09)	0.076^a^
**Moderate**	96 (50.53)	94 (49.47)	
**Disharmonious**	20 (50.00)	20 (50.00)	
**Relationship with father**			
**Harmonious**	553 (49.91)	555 (50.09)	0.988^a^
**Moderate**	96 (50.53)	94 (49.40)	
**Disharmonious**	20 (50.00)	20 (50.00)	
**Relationship with mother**			
**Harmonious**	587 (49.96)	588 (50.00)	0.947^a^
**Moderate**	61 (49.59)	62 (50.41)	
**Disharmonious**	21 (52.50)	19 (47.50)	
**Father’s educational level**			
**Middle school or lower**	418 (51.54)	393 (48.46)	0.074^a^
**High/technical secondary school**	219 (46.3)	254 (53.70)	
**Junior college or higher**	32 (59.26)	22 (40.74)	
**Mother’s educational level**			
**Middle school or lower**	432 (50.59)	422 (49.41)	0.813^a^
**High/technical secondary school**	218 (48.77)	229 (51.23)	
**Junior college or higher**	19 (51.35)	18 (48.65)	
**Parenting style**			
**Democracy**	471 (50.32)	465 (49.68)	0.901^a^
**Autocratic**	122 (48.61)	129 (51.39)	
**Doting**	54 (51.92)	50 (48.08)	
**Disregardful**	22 (46.81)	25 (53.19)	
**Family economic status**			
**Good**	587 (49.96)	588 (50.04)	0.107^a^
**Moderate**	61 (49.59)	62 (50.41)	
**Bad**	21 (52.50)	19 (47.50)	
**Academic achievements**			
**Good**	258 (51.29)	245(48.71)	0.477^a^
**Moderate**	322 (47.56)	355 (52.44)	
**Bad**	89 (56.33)	69 (43.67)	
**Number of close friends**			
**≤2**	201 (52.48)	182 (47.52)	0.787^a^
**3–5**	327 (48.59)	346 (51.41)	
**≥6**	141 (50.00)	141 (50.00)	
**Age**	12.34±1.64	12.39±1.51	0.643^b^

^a^ *P* values were calculated using the chi-square test.

^b^ *P* values were calculated using the rank sum test.

Quantitative data were expressed by “mean ± standard deviation”, while qualitative data were expressed by “n (%)”.

After adjusting the confounding factors, the mixed linear model was fitted, and the results are shown in Table 3. Compared with the control group, the improvement of target focus, emotional control, interpersonal assistance and total mental resilience in the intervention group was significant (*P* < 0.05). No significant difference in score changes were found between primary and secondary schools (*P* > 0.05).

**Table 3:** Comparison of changes in psychological resilience scores between the two groups during follow-up after PSM (n = 1, 338).

Outcome variable	Group	ICC	Baseline	Follow up—Baseline	*P*	*β*	95% *CI*
**Target focus**	Intervention	0.019	17.38 ± 4.31	-0.20 ± 5.28	0.030	-0.813	[-1.544, -0.082]
	Control		17.70 ± 4.52	-1.06 ± 5.00			
**Emotional control**	Intervention	0.005	19.34 ± 4.82	1.11 ± 5.90	<0.001	-1.754	[-2.435, -1.074]
	Control		20.00 ± 4.75	-0.65 ± 5.46			
**Positive cognition**	Intervention	0.023	13.84 ± 3.48	0.24 ± 4.34	0.621	-0.154	[-0.786, 0.477]
	Control		13.81 ± 3.55	-0.65 ± 5.46			
**Family support**	Intervention	0.017	19.96 ± 4.43	0.01 ± 5.47	0.057	-0.725	[-1.472, 0.022)
	Control		20.57 ± 4.48	-0.75 ± 5.29			
**Interpersonal assistance**	Intervention	0.003	19.52 ± 4.73	0.62 ± 5.98	<0.001	-1.701	[-2.399, -1.003]
	Control		20.09 ± 5.08	-1.09 ± 6.00			
**Total resilience**	Intervention	0.034	90.06 ± 13.82	1.77 ± 16.88	<0.001	-4.995	[-7.624, -2.366]
	Control		92.17 ± 14.88	-3.55 ± 15.83			

*P* values were calculated using the mixed linear model.

ICC, based on mixed model analysis, are presented in Table 3 to indicate clustering effects for the investigated outcomes. They were generally low and the highest clustering effects concerned total resilience, which was 0.034.

### Effects of intervention on resilience

In the generalized linear mixed model, independent variables included intervention (peer education/control), age, “relationship between parents”, “relationship with father”, “father’s education level”, “parenting style”, and “family economic status”, with changes in resilience between baseline and final follow-up as the dependent variable and school as a random effect. Table 4 indicates that the intervention was effective for increasing the target focus (*β* = 0.893, SE = 0.282, *P* = 0.002), emotional adjustment (*β* = 1.766, SE = 0.389, *P* < 0.001), interpersonal assistance (*β* = 1.722, SE = 0.716, *P* = 0.016), and total resilience (*β* = 5.391, SE = 1.094, *P* < 0.001), but there were no significant differences in positive cognition and family support (*P* > 0.05).

**Table 4:** The effect of peer education between the intervention group and the control group (n = 1338).

Sample	Resilience	*β*	SE	t	*P*	95% *CI*
**All subjects**	Target focus	0.893	0.282	3.162	0.002	[0.339,1.447]
	Emotional control	1.766	0.389	4.543	<0.001	[1.004,2.529]
	Positive cognition	0.219	0.254	0.862	0.389	[-0.279,0.716]
	Family support	0.737	0.382	1.930	0.054	[-0.012,1.486]
	Interpersonal assistance	1.722	0.716	2.406	0.016	[0.318,3.126]
	Total resilience	5.391	1.094	4.930	<0.001	[3.246,7.536]
**Male**	Target focus	0.882	0.425	2.078	0.038	[0.049,1.716]
	Emotional control	1.935	0.445	4.350	<0.001	[1.061,2.808]
	Positive cognition	0.099	0.336	0.295	0.768	[-0.561,0.760]
	Family support	0.664	0.535	1.242	0.215	[-0.385,1.714]
	Interpersonal assistance	1.276	0.435	2.929	0.004	[0.421,2.131]
	Total resilience	4.889	1.223	3.997	<0.001	[2.488,7.290]
**Female**	Target focus	0.151	0.083	1.823	0.070	[-0.012,0.314]
	Emotional control	1.625	0.439	3.699	<0.001	[0.762,2.488]
	Positive cognition	0.351	0.326	1.077	0.282	[-0.289,0.991]
	Family support	0.052	0.093	0.563	0.574	[-0.131,0.235]
	Interpersonal assistance	2.302	1.060	2.171	0.030	[0.220,4.383]
	Total resilience	6.182	1.333	4.637	<0.001	[3.564,8.800]

Independent variable: Control versus Intervention.

Dependent variable: the difference of resilience between baseline and final follow-up.

Confounding factors including "relationship between parents", "relationship with father", "father’s educational level", "parenting style", "family economic status", and age.

Table 4 shows sex differences in the effectiveness of the intervention. For boys, target focus (*β* = 0.882, SE = 0.425, *P* = 0.038), emotional adjustment (*β* = 1.935, SE = 0.445, *P* < 0.001), interpersonal assistance (*β* = 1.276, SE = 0.435, *P* = 0.004), and total resilience (*β* = 4.889, SE = 1.223, *P* < 0.001) were increased significantly. However, for girls, there were significant improvements only in emotional adjustment (*β* = 1.625, SE = 0.439, *P* < 0.001), interpersonal assistance (*β* = 2.302, SE = 1.060, *P* = 0.030), and total resilience (*β* = 6.182, SE = 1.333, *P* < 0.001).

## Discussion

To date, a growing body of research has shown a sharp rise in the number of children and adolescents with mental health problems. Poor mental health among children and adolescents usually leads to a more positive attitude toward suicide, which in turn promotes greater suicidal ideation [50–52]. From the perspective of positive psychology, better resilience can help individuals overcome and work through negative stresses. In previous studies of intervention, a large body of studies increased the resilience of children and adolescents by improving coping strategies, cognitive restructure, and resilience factors [53], but adolescents are a special period—adolescence—facing various physiological and psychological changes. Therefore, intervention based on adolescent health education is necessary and innovative. Then, previous intervention models were built on school and family circumstances, considering parents and teachers as carriers of increasing resilience [53]. However, adolescents spend more time with peers than with parents during adolescence, so it is worth trying to utilize peer education to improve the resilience of adolescents.

Our study found that peer education based on adolescent health education could improve the target focus, emotional adjustment, and interpersonal assistance of adolescents, which is consistent with previous research [54]. Regarding target focus, the research conducted by Jacquline Schwab and colleagues [55] implicated that the hormonal changes associated with sex development have profound influences on self-perceived competence, which was referred to as the efficacy or skillfulness of accomplishing age-appropriate tasks to fulfil personal goals and demands successfully. Therefore, delivering positive adolescence-related knowledge, attitudes, and behaviors to subjects might offset the negative effect of sex maturation. Additionally, because of higher academic pressure, adolescents are prone to engage in less physical activity, which is associated with self-efficacy [56, 57]. There is a positive correlation between health literacy and self-efficacy [58], and self-efficacy and goal setting can help them be more purposeful in planning their activities and participating in a normal lifestyle [59]. Moreover, it has been found that in the United States, self-efficacy is considered a protective factor for increased resilience [60]. Cultivating healthy lifestyles, including a healthy diet and reasonable exercise, could enhance self-efficacy to cope with problems and improve the target focus of personal resilience. Emotional intelligence is one of the most important topics for adolescent mental health professionals [61]. With respect to emotional adjustment, Lauren and colleagues [16] found that the increased gap between physiological development and psychological development due to early pubertal timing was a risk of rumination that was characterized by the tendency to pay close attention to one’s dysphoric mood passively and repetitively, as well as its meanings and consequence; furthermore, some studies have indicated that reasonable exercise can overcome emotion regulation difficulties [54]. Our intervention based on adolescent health education and a healthy lifestyle can decrease the gap and improve emotion regulation difficulties. In terms of interpersonal assistance, some studies have shown that peer-to-peer teaching can enhance communication skills [62]. In our study, a knowledge quiz game, group discussion, sitcom performance, and poster design can increase the opportunity for communication with peers, raising team awareness. These benefits may be the reason for increased interpersonal assistance. In particular, no significant improvement was found in positive cognition and family support in our study. The reasons may be that our intervention mainly involves adolescent health education and positive coping styles, with less positive cognition toward difficulties and family support, which also suggests that more attention should be devoted to these two dimensions.

In terms of total resilience, we found that, in the absence of intervention measures, the control group of students’ resilience decreased, while the resilience of the intervention group significantly improved after peer education. In other words, peer education intervention based on adolescent health education could weaken the negative impacts of adolescent upheavals on the resilience of adolescents and even reverse the developmental trajectory of young people’s mental resilience. This further demonstrates that peer education of resilience for children and adolescents is successful by taking physiological knowledge, mental health education and healthy lifestyle as targeted interventions. Adolescence serves as a transitional period and can be "leveraged to encourage positive development trajectories" through interventions [63], such as peer intervention, individual and group psychological interventions, and school and community projects [12, 64], to support adolescent resilience.

Gender differences are also a cause for concern. Significant gender differences have been shown to exist in potentially traumatic events and subsequent posttraumatic stress disorder [65], and other studies have found that women are more likely to develop mental or physical problems in response to life stresses or potentially traumatic events [66–68]. In other words, the psychological problems of girls are more difficult to intervene than those of men. Our results also indicate gender differences in the effectiveness of resilience interventions, with more effectiveness for males than for females. The intervention effect of male students is mainly reflected in goal focus, emotion control, interpersonal assistance and total resilience, while that of female students is mainly reflected in emotion control, interpersonal assistance, and total resilience. It follows that the intervention of goal focus is easier for men than for women.

The effect of clustering must be taken into account in the results. The intra-cluster correlation coefficient (ICC) is the proportion of the total variance of the results that can be explained by inter-cluster differences, and is often used to estimate the clustering effect of an experiment [41]. In this study, the ICC of the main outcome variables were all less than 0.05. On the one hand, the ICC value is smaller than the ICC size assumed when calculating the sample size, indicating that the actual sample size of this study meets the requirements. On the other hand, the small ICC indicates that the difference in outcome between the intervention group and the control group is mainly caused by peer education, rather than inter-group differences, and peer education is effective in the intervention of mental resilience. In addition, it can provide information for future cluster trials in similar environment.

Many mental health disorders emerge in late childhood and early adolescence and impose a burden of these disorders on young people and later in life [69]. In 2019, China’s Ministry of Education and 10 other government departments launched a joint action plan to pay special attention to the mental health of children and adolescents. According to the action plan, schools at all levels and of all types should establish psychological service platforms to provide mental health services for students by 2022 [2]. Schools play an important role in the transition of children and adolescents to adulthood. Although many schools provide basic health information and education to adolescents, teaching methods seem ineffective due to a number of factors, including culture, religion and belief. The results of this study emphasize that policy makers should attach importance to supporting the implementation and development of mental health education in schools when implementing health care reform, and recognize that peers are important factors affecting adolescents’ positive mental health. Advocate for the positive development of adolescents by engaging them in engaging peer activities, rather than just health education lectures and questions. Adolescence is a time of life when many risks lurk, but because of adolescents’ curiosity and interest in learning about themselves, it also offers great opportunities to achieve sustained health and well-being through education and prevention efforts [70]. By combining traditional education with peer education to complement each other’s advantages, it is expected to better enhance the positive mental health of adolescents in China.

### Strengths and limitations

First of all, to our knowledge, no studies have thoroughly explored the effect of peer education on the psychological resilience of adolescents. Secondly, this study is a clustered randomized controlled trial, which can more effectively control the contamination between the intervention group and the control group in the school-based intervention.

Despite the potential of these findings to verify the effect of peer education based on adolescent health education on the resilience of adolescents, it is important to acknowledge certain limitations of the study. On the one hand, our sample is only from Qijiang District, Chongqing. The representativeness of the sample is limited and cannot represent all children and adolescents. Moreover, the number of clusters in this study is insufficient, which may lead to inaccurate estimation of intervention effect. Therefore, in future studies, the sample should be expanded to improve the sample representativeness. On the other hand, since the inclusion and exclusion criteria were set at the beginning of the study, and strict condition control was implemented during the intervention and follow-up process, the results may not necessarily represent the real adolescent intervention environment.

## Conclusions

Our peer education based on adolescent health education can significantly improve target focus, emotional adjustment, interpersonal assistance and total resilience, and there are gender differences in the intervention effect. In addition, more attention should be devoted to positive cognition toward difficulties and family support to perfectly increase the resilience of adolescents.

## Supporting information

S1 File The effect of peer education training on pubertal knowledge and attitudes for boys and girls (n = 76). After peer education training, the training effect results showed that the correct rate of adolescent-related health knowledge and attitude scores of peer educators were significantly improved.
(DOCX)

S1 Table Peer education content arrangement. Psychological health education includes the process of psychological development during adolescence and the treatment of psychological problems such as tension, anxiety and conflicts with parents, teachers, and peers; a healthy lifestyle involves a balanced diet, reasonable exercise, and good sleep.
(DOCX)

S1 Text The protocol of this study. (DOCX)
