# Supplementary material for: The effect of peer education based on adolescent health education on the resilience of children and adolescents: A cluster randomized controlled trial
Source: PLoS One. 2022 Feb 2;17(2):e0263012. doi: 10.1371/journal.pone.0263012 (PMC8809556; doi:10.1371/journal.pone.0263012)
Supplement: S1 File — After peer education training, the training effect results showed that the correct rate of adolescent-related health knowledge and attitude scores of peer educators were significantly improved. (DOCX) [file pone.0263012.s001.docx]

After peer educator training, the results of the effectiveness of training showed that the correct rate of adolescent-related health knowledge and attitude improved significantly (see Supplementary Tables 1-3).

| Supplementary Table 1 The effect of peer educator training on pubertal knowledge for boys (*n*=38) | | | | |
| --- | --- | --- | --- | --- |
| Knowledge | Before (%) | After (%) |  | *P* |
| Q1 | 28 (73.68) | 37 (97.37) | 7.111 | 0.004 |
| Q2 | 26 (68.42) | 38 (100.00) | 10.083 | <0.001 |
| Q3 | 32 (84.21) | 38 (100.00) | 4.167 | 0.031 |
| Q4 | 32 (84.21) | 38 (100.00) | 4.167 | 0.031 |
| Q5 | 26 (68.42) | 38 (100.00) | 10.083 | <0.001 |
| Q6 | 33 (86.84) | 38 (100.00) | 3.200 | 0.062 |
| Q7 | 35 (92.11) | 38 (100.00) | 1.333 | 0.250 |
| Q8 | 33 (86.84) | 38 (100.00) | 3.200 | 0.062 |
| Q9 | 24 (63.16) | 38 (100.00) | 12.071 | <0.001 |
| Q10 | 18 (47.37) | 31 (81.58) | 7.579 | 0.004 |
| Q11 | 7 (18.42) | 35 (92.11) | 26.036 | <0.001 |
| Q12 | 19 (50.00) | 38 (100.00) | 17.053 | <0.001 |
| Q13 | 27 (71.05) | 38 (100.00) | 9.091 | 0.001 |
| Q14 | 27 (71.05) | 38 (100.00) | 9.091 | 0.001 |
| Q15 | 34 (89.47) | 38 (100.00) | 2.250 | 0.125 |
| Q16 | 10 (26.32) | 37 (97.37) | 25.037 | <0.001 |
| Q17 | 24 (63.16) | 38 (100.00) | 12.071 | <0.001 |
| Q18 | 23 (60.53) | 29 (76.32) | 2.083 | 0.146 |
| Q19 | 22 (57.89) | 36 (94.74) | 10.562 | 0.001 |
| Q20 | 33 (86.84) | 37 (97.37) | 2.250 | 0.125 |
| Q21 | 30 (78.59) | 38 (100.00) | 6.125 | 0.008 |
| Q22 | 21 (55.26) | 38 (100.00) | 15.059 | <0.001 |
| Q23 | 31 (81.58) | 36 (94.74) | 3.200 | 0.062 |

| Supplementary Table 2 The effect of peer educator training on pubertal knowledge for girls（n=38） | | | | |
| --- | --- | --- | --- | --- |
| Knowledge | Before (%) | After (%) |  | *P* |
| Q1 | 30 (78.59) | 35 (92.11) | 1.788 | 0.180 |
| Q2 | 28 (73.68) | 37 (97.37) | 5.818 | 0.012 |
| Q3 | 30 (78.95) | 38 (100.00) | 6.125 | 0.008 |
| Q4 | 26 (68.42) | 36 (94.74) | 8.100 | 0.002 |
| Q5 | 35 (92.11) | 38 (100.00) | 1.333 | 0.250 |
| Q6 | 33 (86.84) | 38 (100.00) | 3.200 | 0.062 |
| Q7 | 24 (63.16) | 38 (100.00) | 12.071 | <0.001 |
| Q8 | 33 (86.84) | 37 (97.37) | 3.200 | 0.062 |
| Q9 | 26 (68.42) | 29 (76.32) | 0.364 | 0.549 |
| Q10 | 27 (71.05) | 38 (100.00) | 9.091 | 0.001 |
| Q11 | 34 (89.47) | 37 (97.37) | 0.800 | 0.375 |
| Q12 | 29 (76.32) | 38 (100.00) | 7.111 | 0.004 |
| Q13 | 25 (65.79) | 38 (100.00) | 11.077 | <0.001 |
| Q14 | 32 (84.21) | 38 (100.00) | 4.167 | 0.031 |
| Q15 | 15 (39.47) | 38 (100.00) | 21.043 | <0.001 |
| Q16 | 22 (57.89) | 37 (97.37) | 11.529 | <0.001 |
| Q17 | 25 (65.79) | 36 (94.74) | 9,091 | 0.001 |
| Q18 | 16 (42.11) | 38 (100.00) | 20.045 | <0.001 |
| Q19 | 29 (76.32) | 38 (100.00) | 7.111 | 0.004 |
| Q20 | 29 (76.32) | 38 (100.00) | 7.111 | 0.004 |
| Q21 | 27 (71.05) | 36 (94.74) | 7.111 | 0.004 |
| Q22 | 23 (60.53) | 37 (97.37) | 12.071 | <0.001 |
| Q23 | 19 (50.00) | 37 (97.37) | 16.056 | <0.001 |

| Supplementary Table 3 The effect of peer educator training on pubertal attitude for boys and girls（*n*=76） | | | | |
| --- | --- | --- | --- | --- |
| Attitude | Before ($\bar{\chi}\pm s$) | After ($\bar{\chi}\pm s$) | t | *P* |
| A1 | 4.74±0.60 | 4.93±0.34 | -3.174 | 0.002 |
| A2 | 4.54±0.77 | 4.97±0.16 | -5.018 | <0.001 |
| A3 | 4.82±0.45 | 5.00±0.00 | -3.542 | 0.001 |
| A4 | 4.17±1.12 | 4.89±0.53 | -5.721 | <0.001 |
| A5 | 3.40±1.28 | 4.59±1.01 | -6.761 | <0.001 |
| A6 | 4.79±0.60 | 4.96±0.26 | -2.496 | 0.015 |
| A7 | 4.81±0.56 | 4.97±0.16 | -2.429 | 0.018 |
| A8 | 3.93±1.25 | 4.93±0.25 | -7.134 | <0.001 |
| A9 | 4.55±0.81 | 4.75±0.80 | -2.066 | 0.042 |
| A10 | 4.64±0.67 | 4.86±0.61 | -2.150 | 0.035 |
