# Supplementary material for: The effect of peer education based on adolescent health education on the resilience of children and adolescents: A cluster randomized controlled trial
Source: PLoS One. 2022 Feb 2;17(2):e0263012. doi: 10.1371/journal.pone.0263012 (PMC8809556; doi:10.1371/journal.pone.0263012)
Supplement: S1 Table — Psychological health education includes the process of psychological development during adolescence and the treatment of psychological problems such as tension, anxiety and conflicts with parents, teachers, and peers; a healthy lifestyle involves a balanced diet, reasonable exercise, and good sleep. (DOCX) [file pone.0263012.s002.docx]

Psychological health education includes the process of psychological development during adolescence and the treatment of psychological problems such as tension, anxiety and conflicts with parents, teachers, and peers; a healthy lifestyle involves a balanced diet, reasonable exercise, and good sleep.

| S1 Table Peer education content arrangement |
| --- |
| **Physical health education** |
| **Chapter 1** Entering Adolescence  What is the adolescence  Adolescent staging (early adolescence, mid-adolescence, late adolescence) and the characteristics of development in each adolescent stage  The reason of adolescent changes |
| **Chapter 2** Changes in Adolescence  Development of secondary sexual characteristics in boys and girls  Precautions during voice change period  Breast care and precautions during breast development |
| **Chapter 3** Whelk in Adolescence  What is the whelk  The reasons of occurrence of whelk during  How to treat whelk |
| **Chapter 4** Spermatorrhea  What is the spermatorrhea  The reasons of occurrence of spermatorrhea  Measures taken after the occurrence of nocturnal emission |
| **Chapter 5** Menstruation  What is the menarche  Normal menstrual cycle  Normal blood volume, blood color and morphology of menstruation  Taboo during menstruation  Health care during menstruation  What is the dysmenorrhea  How to relive dysmenorrhea |
| **Psychological health education** |
| **Chapter 6** Mental Health  Psychological characteristics of adolescents during adolescence  Debugging and coping skills of adolescent psychological problems  How to deal with the conflicts with parents, teachers and peers  How to deal with heterosexual relationships |
| **Chapter 7** Mental resilience  How to achieve our goals  How to build confidence  How to identify healthy psychology and abnormal psychology  How to deal with setbacks and pressures |
| **Health lifestyle** |
| **Chapter 8** Health lifestyle  The balanced diet  The reasonable exercise  No smoking and drinking  Keeping good sleeping |
