# Supplementary material for: The effect of peer education based on adolescent health education on the resilience of children and adolescents: A cluster randomized controlled trial
Source: PLoS One. 2022 Feb 2;17(2):e0263012. doi: 10.1371/journal.pone.0263012 (PMC8809556; doi:10.1371/journal.pone.0263012)
Supplement: S1 Text — (DOCX) [file pone.0263012.s003.docx]

**Research protocol: part 1**

**The effect of peer education** **based on adolescent health education on** **resilience of children and adolescents: study protocol for a cluster randomized controlled trial**

Yinshuang Tang^1^ M.D, Hua Diao^1^ M.D, Feng Jin^1^ M.D, Yang Pu^1^ M.D, Hong Wang^1,*^ M.D

^1^ School of Public Health and Management, Chongqing Medical University, Research Center for Medicine and Social Development, Collaborative Innovation Center of Social Risks Governance in Health, Chongqing Medical University, Chongqing, China.

^*^ corresponding author

E-mail:[wangh111111@aliyun.com](mailto:wangh111111@aliyun.com) (HW) Telephone number:18996351672

E-mail:[1403768683@qq.com](mailto:1403768683@qq.com) (Yinshuang Tang)

[diaohwlkq@aliyun.com](mailto:diaohwlkq@aliyun.com) (Hua Diao)

[1272039330@qq.com](mailto:1272039330@qq.com) (Feng Jin)

[904065192@qq.com](mailto:904065192@qq.com) (Yang Pu)

### **Project summary**

**Background:** A increasing number of children and adolescents have mental health problems, but resilience is a protective factor of these problems. Therefore, aim of the study is to verify the effect of peer education based on adolescent health education on resilience.

**Method:** A cluster randomized controlled trial will be conducted. Subjects will be divided into an intervention group and a control group. The intervention group will receive 1 year of peer education, and adolescent physiological knowledge, mental health education and healthy lifestyle will be targeted intervention content, while the control group will not receive any intervention. A Resilience Scale for Chinese Adolescent by Yueqin Hu and a self-designed basic information questionnaire will be used to collect data. Chi-square test and rank-sum test will used to compare the differences between the two groups, and a generalized linear mixed model will be used to verify the effect of peer education on adolescent resilience. The significance will be set at P < 0.05.

**Outcomes:** We believe that the increase of mental resilience scores in the intervention group will be greater than that in the control group. Peer education based on adolescent health education will effectively improve adolescent's mental resilience.

**Keywords**: Resilience; Peer education; Adolescents; Mental health

### **Background**

In China, with the rapid development of social economy and the change of social structure in recent decades, social pressure and competition have gradually increased. For adolescents, higher academic requirements and psychological pressure lead to the increase of negative emotions, and mental health problems are becoming increasingly prominent [1-2]. It is estimated that more than 100 million of China's 1.3 billion people suffer from mental disorders, of which about 16 million are seriously ill [3]. A national epidemiological study has found that 15% of children in China suffer from mental health problems, and the prevalence of anxiety and other diseases is rising [4].

With the development of positive psychology, researchers have devoted more attention to the positive effect of negative events on the mental health of adolescents [5-6]. In other words, not all individuals in adversity will experience negative outcomes such as anxiety and depression, and some individuals will instead experience better positive outcomes as a result of negative circumstances [7]. Therefore, some experts have proposed the term “resilience”, which is defined as the ability to withstand and recover from adverse environments in an effective manner [8]. As one of the protective factors of mental health problems [9], it can protect individuals from negative psychological problems such as stress and anxiety, and promote high self-esteem to reduce depressive symptoms [10-11]. Resilience is influenced by a large number of influencing factors, including protective factors such as harmonious family relationships, friendly peer relationships, and positive coping skills [12-13].

Adolescents are at a critical point in the transition from child to adults, involving a variety of physiological, psychological, and social function upheavals (e.g., genital development and pubic hair growth) that are stressful events for them [14-15]. A robust body of research indicates that pubertal status is a key predictor of various internalizing and externalizing problems for adolescents that influence the resilience of adolescents [16-17]. For instance, a study with 1,420 subjects, conducted by Angold et al. [18], showed that girls at Tanner 3 were over three times more likely to contact depressive disorders than girls in the earlier Tanner stages, independent of the age at which they entered stage 3. A growing body of literature implies that pubertal timing of adolescents occurs earlier [19-20]. Earlier pubertal timing will increase the risk of internalizing and externalizing problems, aggravating the negative influence of adolescent changes on the mental health of adolescents [21-23]. For example, Mendle found that earlier pubertal timing had a significant association with depression and anxiety [24]. Therefore, we consider improving resilience by increasing positive adolescence-related knowledge, attitudes and behaviors.

The Healthy China Action (2019-2030) plan explicitly mentions initiatives to promote mental health and student health [25]. However, although the government has introduced important policies and paid more attention to mental health, more research and guidance are needed to build an interrelated and efficient mental health system [2]. A large number of previous studies have found that traditional health education in school classroom can effectively promote mental health such as resilience, anxiety and depression [26-28], and health risk behaviors such as smoking, alcohol abuse and unintentional injury [29-31], but the effect is incomplete and often short-term. Schools are key places to improve adolescent health, but classroom education is also challenging as schools increasingly focus on indicators of academic achievement [32]. After puberty, adolescents spend more time with their peers and have a stronger sense of identity with their peers [33], which suggests that peer education may be an effective way to improve the mental health of Chinese adolescents.

Peer education is defined as “sharing experiences and learning among those of a similar age, living environment, and culture with something” [34], which is based on social cognitive theory [35] that shows that the interactions and observations of others can impact the behavior and attitude of individuals. A large number of studies have shown that peer education is extremely effective in some domains, such as prevention of chronic diseases and dissemination of sexual knowledge [34, 36-37]. This suggests that it is of great practical significance to adopt the method of peer education to intervene the resilience of Chinese adolescents. In most previous clinical trials, participants were randomized as individuals to receive different interventions. However, it is worth noting that sometimes individual assignment is not possible or desirable. Indeed, many educational evaluations are conducted in naturally occurring clusters (such as classes or schools), and the only practical way to conduct a randomized controlled trial (RCT) is to use cluster assignment. Cluster randomization is often used to avoid contamination between those who receive intervention and those who do not [38]. Using clusters rather than individuals as random units has proven to be a more efficient and economical option [39].

**Study goals and objectives**

In general, the aim of study is to conduct a cluster randomized controlled trial (cluster RCT) to verify the intervention effect of peer education based on adolescence-related knowledge, attitudes, and behaviors on adolescents' psychological resilience.

In detail, through the investigation of adolescent life quality and its influencing factors, to understand the quality of life of this population and its influencing factors, and try to carry out peer psychological mutual assistance intervention, in order to improve the awareness rate of adolescent health knowledge of this population, help them to establish a correct view of health, form a good health behavior and life style. Finally achieve the purpose of promoting their physical and mental health and improving their quality of life, and explore a scientific and reasonable effective way to improve the quality of life of adolescence.

**Methods**

### **Study design**

A cluster randomized controlled trial will be conducted in 4 schools (2 primary schools and 2 middle schools) with similar conditions in Qijiang District of Chongqing. According to the school level, the project quality manager will used a computer random number generator to randomly divide the selected four schools into an intervention group with peer education intervention and a control group without any intervention (blank control group), with a ratio of 1:1. At follow-up, the study group investigators will blinded to group assignment.

The inclusion criterion include that subjects belong to grades 4 to 5 in primary schools and grades 7 to 8 in middle schools, and can complete questionnaire independently. The exclusion criterion include that subjects with intellectual disabilities. The investigation is expected to take a year and a half. Considering that students in grade 6 and grade 9 will lose due to enter a higher school, so will not be included in the study. Students who meet all the inclusion criteria and none of the exclusion criteria will have the opportunity to participate in the study and will receive informed consent orally and in writing.

In December 2017, all students will take approximately 40 minutes to complete the baseline questionnaire. In April 2,018, peer educator training will be conducted by experts in health of children and adolescents. After 1-year intervention, the final follow-up will be performed in May 2,019. Figure 1 shows a diagram with the different phases of the study. The study was approved by Biomedical Ethics Committee of Peking University (IRB 00001052–13,034) and the ethical committee of the Chongqing Medical University and written inform consent was obtained from students and their parents before investigation in the research.


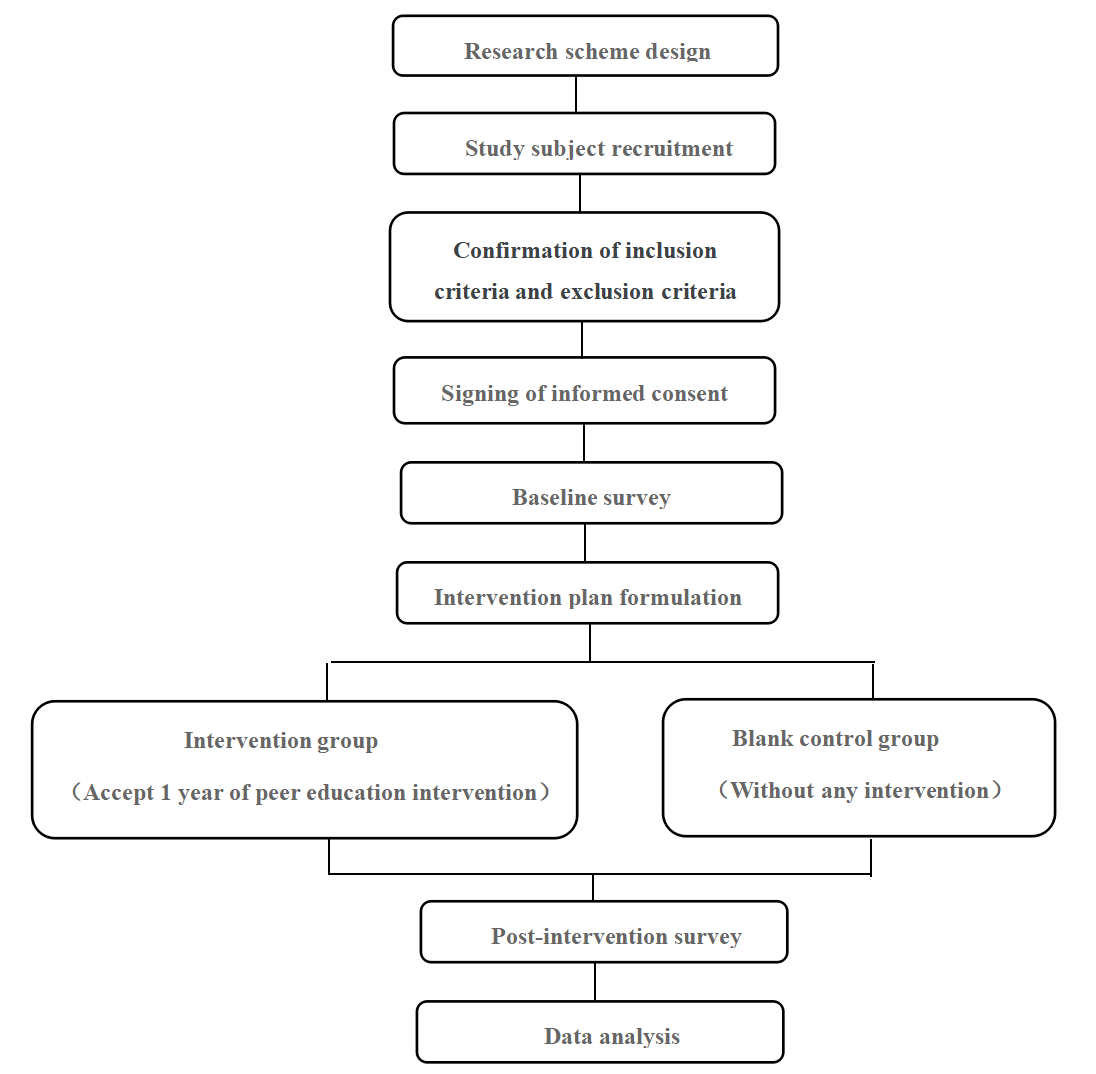


Fig. 1 Flow of the participants throughout the study

**Sample size**

For superiority trials comparing the means of the two groups, the following formula was used to calculate the sample size [40-41]:

$n=\left[\frac{(U_{1-\alpha}+U_{1-\beta})\sigma}{\delta}\right]^{2}\times\frac{1+C}{C}$ (1)

In formula (1), σ is defined as standard deviation; δ is referred to as a clinically meaningful low or high limit that is the width of the 95% confidence interval equally; c is the ratio of sample cases between intervention group and control group that is equal to 1 in this study. In our research, α and β were 0.05 and 0.10, respectively, so U1-α and U1-β were 1.64 and 1.28, respectively. Pre-surveys revealed that σ and δ are 14.85 and 3.14, respectively. Therefore, a sample size of at least 275 was calculated for both groups.

$DE=1+\text{（}m-1)\rho$ (2)

Considering that this study was a cluster RCT, the sample size should be increased through design effect (DE) to ensure the efficiency of the test [42-43]. When the average cluster size (m) is 40 and the intra-cluster correlation coefficient (ICC) is 0.05, the calculated DE is 2.95. The DE was multiplied by the sample size without clustering effect (n), resulting in a sample size of 811 students in each group and a cluster number of 20 in each group.

**Intervention: peer education involving adolescent health education**

In the intervention group schools, negotiating with head teachers in every class, 4 excellent, responsible and well-communicated students, including 2 boys and 2 girls, were selected as peer educators who served on the class committee, actively participated in extracurricular activities and had activity organization experiences. Peer education training seminars were organized by members of the study group to provide peer educators with targeted training on adolescent physiology, mental health and healthy lifestyle. The training teacher of each group will be composed of a professor from Chongqing Medical University and 3 graduate students from the research group. The training will take the form of intensive teaching, group discussions and knowledge competitions. The training content will be divided into four parts: adolescent physical health knowledge, adolescent mental health knowledge, healthy behavior and lifestyle, and peer education knowledge and skills. Each training content lasts about 1 hour. After the training of 76 peer educators, the effectiveness of the training will be tested by analyzing the changes in the scores of adolescents' health knowledge, attitudes and healthy lifestyle items. Make peer educators' knowledge mastery reach 80% or above to ensure their ability to carry out peer education activities. Regarding the forms of peer education, knowledge quiz game, group discussion, performing sitcom, self-design poster exhibition and so on will be used to conduct activities, with provision of knowledge quiz software, group discussion cases and analysis results, role-playing scripts, and adolescence-related health education slides. Peer educators will use class meetings or spare time to conduct activities among peers and will be required to record key information about each activity, such as the number of participants, activity context, participant satisfaction and suggestions. As well, educators are expected to conduct activities at least twice a month. In October 2018, we will conduct intermediate training to strengthen relevant knowledge and teach them how to deal with stress and negative events. During 1-year intervention, we will perform supervision twice per semester to evaluate the process of peer education activities. The forms of supervision include watching peer educators carry out activities in class on the spot, checking the activity record manual, collecting the materials of previous activities, and providing reference suggestions for peer educators to better carry out activities.

With the respect of content of peer education sessions, based on the "Six Strategies Training Program" by Henderson and Milstein [44] and the key life period in that adolescents are transitioning from child to adult, physiological knowledge, psychological health education, and health lifestyles were included as the targeted intervention content. Physiological health education involves growth spurts, development of secondary sexual characteristics, acne treatment, treatment of breast development, menstruation, dysmenorrhea for girls, treatment of beard growth and seminal emission, and cleaning of the private parts. Psychological health education includes process of psychological development during adolescence, and treatment of psychological problems such as tension, anxiety and conflicts with parents, teachers, and peers; healthy lifestyle involves a balanced diet, reasonable exercise, and keeping good sleep. See Supplementary Table1.

| Table 1 Peer education content arrangement |
| --- |
| **Physical health education** |
| **Chapter 1** Entering Adolescence  What is the adolescence  Adolescent staging (early adolescence, mid-adolescence, late adolescence) and the characteristics of development in each adolescent stage  The reason of adolescent changes |
| **Chapter 2** Changes in Adolescence  Development of secondary sexual characteristics in boys and girls  Precautions during voice change period  Breast care and precautions during breast development |
| **Chapter 3** Whelk in Adolescence  What is the whelk  The reasons of occurrence of whelk during  How to treat whelk |
| **Chapter 4** Spermatorrhea  What is the spermatorrhea  The reasons of occurrence of spermatorrhea  Measures taken after the occurrence of nocturnal emission |
| **Chapter 5** Menstruation  What is the menarche  Normal menstrual cycle  Normal blood volume, blood color and morphology of menstruation  Taboo during menstruation  Health care during menstruation  What is the dysmenorrhea  How to relive dysmenorrhea |
| **Psychological health education** |
| **Chapter 6** Mental Health  Psychological characteristics of adolescents during adolescence  Debugging and coping skills of adolescent psychological problems  How to deal with the conflicts with parents, teachers and peers  How to deal with heterosexual relationships |
| **Chapter 7** Mental resilience  How to achieve our goals  How to build confidence  How to identify healthy psychology and abnormal psychology  How to deal with setbacks and pressures |
| **Health lifestyle** |
| **Chapter 8** Health lifestyle  The balanced diet  The reasonable exercise  No smoking and drinking  Keeping good sleeping |

**Measurements and outcomes**

Psychological resilience was assessed through Resilience Scale for Chinese Adolescents (RSCA) by Yueqin Hu [45] according to Chinese culture, involving target focus, emotional adjustment, positive cognition, family support and interpersonal assistance. This scale contains 27 items and each item utilizes the 5-point scoring method. The principles of assignment are as follows, positive entries have 5 options: totally inconsistent (1 point), comparatively inconsistent (2 points), unclear (3 points), comparatively consistent (4 points), fully consistent (5 points); reverse entries are counted in reverse: totally inconsistent (5 point), comparatively inconsistent (4 points), unclear (3 points), comparatively consistent (2 points), fully consistent (1 points). The total score range is between 27 and 135, with a higher score indicates a higher level of psychological resilience. We test the reliability of this scale in our research sample, with the resultant Cronbach's alpha of target focus, emotional control, positive cognition, family support, interpersonal assistance, and the overall scale being 0.765, 0.648, 0.709, 0.590, 0.666 and 0.818, respectively. The mental resilience scores of the respondents will be investigated before and after the intervention.

According to the influencing factors of resilience [46-47], following socio-demographic characteristics will be collected through self-designed questionnaires during the baseline survey: age (continuous variable); participants’ education level (primary school/middle school); sex (male/female); whether the individual is an only-child (Yes/No); relationship between parents and with parents (disharmonious/moderate/harmonious) linked to the number of conflicts; parents’ education level (junior high school or lower/senior high school and technical secondary school/college or higher); family economical status (good/moderate/bad); academic achievements (good/moderate/bad); the number of close friends (≤2/3-5/≥6); and parenting style (democratic/autocratic/doting/disregardful).

### **Safety considerations**

All investigations in this study will be carried out in the form of questionnaires and will not involve any invasive tests that may cause harm to the subjects. We pay great attention to the individual wishes of the research subjects, and informed consent of the students and their parents will be obtained before each questionnaire survey. During the implementation of the project, we will keep the relevant information of the research object strictly confidential.

### **Follow-up**

This study plans to implement a one-year peer education intervention for the intervention group. Peer education activities include physical knowledge, mental health education and healthy lifestyle knowledge. Peer education activities will be supervised by psychologists and study members during the intervention. The forms of activity supervision include online contact, on-site observation and activity data collection. During the intervention and supervision of the project, we will pay close attention to the psychological status of the subjects and deal with possible adverse events in a timely manner. After the intervention, all participants will be followed up in the form of questionnaires by project team members.

### **Data management and statistical analysis**

EpiData 3.1 will be used for data input, SAS 9.4 and SPSS24.0 will be used for statistical analysis. The quantitative data will be expressed by mean and standard deviation, and the qualitative data will be expressed by number of cases and percentage. Propensity Score Matching (PSM) will be used for data matching if baseline information differed significantly between the intervention and control groups. T test and one-way ANOVA will be used for the difference between groups of continuous variables with normal distribution, and rank sum test will be used for the difference between groups of continuous variables with non-normal distribution. Chi-square test or Fisher's exact test will be used for differences between groups of categorical variables. After determining the changes between baseline and final follow-up, the effectiveness of peer education intervention on adolescent resilience will be tested using a generalized linear mixed model (GLMM), with the change value of resilience score as the dependent variable and the intervention style and other unmatched inter-group imbalance factors as independent variables. ICC was calculated to explain the clustering effect. ICC will be calculated to explain the clustering effect. The value of ɑ is equal to 0.05.

### **Quality assurance**

In the design of the study, experts in epidemiology, statistics, child and adolescent health, psychology and pedagogy were consulted extensively, and the plan was formulated in combination with the discussion of the research group. Prior to the investigation, all investigators will undergo standardized training to ensure the quality of the investigation. During the survey, the investigator will explain the matters needing attention in questionnaire filling to the research subjects and guide the students to complete the questionnaire filling. The questionnaire will be checked and collected on the spot, and questionnaires with obvious logical mistakes and missing items will be returned to students for timely revision. After the investigation, all survey data will be recorded and checked by two investigators with EpiData 3.1 software. The subsequent processing of outliers and statistical analysis of the data will be supervised by epidemiologists and statistical experts.

### **Expected outcomes of the study**

The expected outcomes of this study contribute to the improvement of peer education based on adolescent health education effectiveness as a tool to improve the quality of life of adolescent children.

Through the training of excellent peer educators and excellent graduate talent team, it will serve for the establishment of the peer health education model in adolescence. To develop and transform the direction of decision-making consultation, to provide decision-making consultation for national and local education administrative departments, medical and health departments and other relevant departments, and to bring adolescent health education into the field of school health services. Transform to socialization, create a powerful social environment for the healthy growth of adolescent children, and serve to promote the healthy growth of adolescent children both physically and mentally and improve their quality of life.

### **Duration of the project**

Before the implementation of the project, the research design was completed by extensively consulting domestic and foreign literature, consulting experts in relevant fields and discussing with members of the research group. In December 2,017, all participants will complete a 40-minute baseline questionnaire. From January to March 2018, baseline survey data processing, peer educator selection and intervention materials preparation are expected to be carried out. In April 2,018, peer educator training will be conducted by experts in health of children and adolescents. In the process of peer education, psychological experts and members of the research group supervise the activities. In October 2018, intermediate training will be conducted for peer educators. After 1-year intervention, the final follow-up will be performed in May 2,019. The specific process is shown in Figure 2.


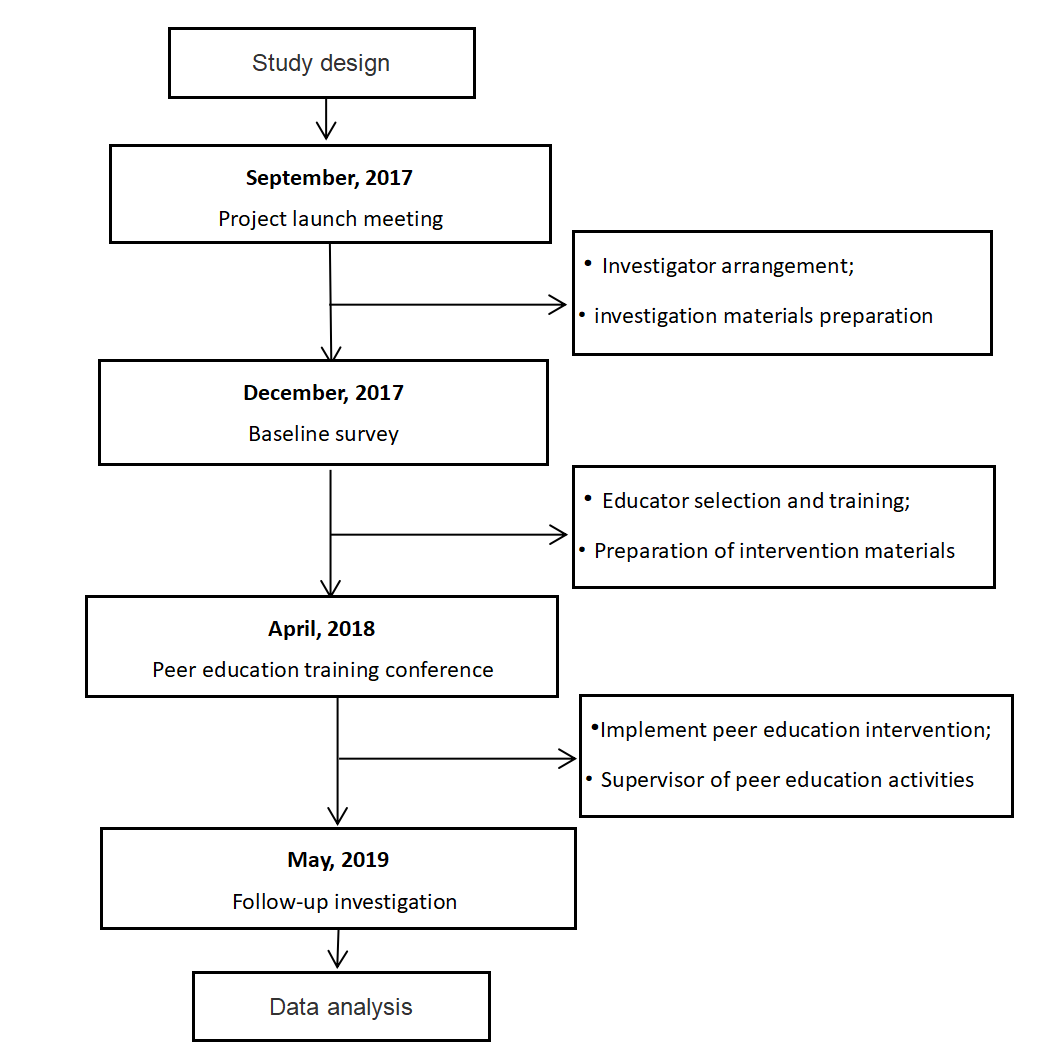


Fig. 2 Flow of the project development process

### **Problems anticipated**

Selecting and training qualified peer educators is a key problem to be solved in this research project. First of all, it is necessary to develop detailed training plans and programs for peer psychological mutual assistance, and to compile operational training materials for peer educators and corresponding publicity materials. Secondly, strive for the strong support of local leaders, school leaders and teachers and the active cooperation of students, mobilizing the active participation and enthusiasm of the target population, according to the selection process and requirements of peer educators, choose appropriate peer educators, and continuous participatory training to improve their ability of health education and skills.

### **Project management**

YT and HD were contributed equally in conception and design, literature search, data acquisition, data analysis, drafting of the initial manuscript and revising it critically for important intellectual content. HW were involved in data acquisition, data analysis and editing the manuscript. FJ, and YP participate in collection of data.

### **Ethical approval and consent to participate**

The study was approved by Biomedical Ethics Committee of Peking University (IRB 00001052–13,034) and the ethical committee of the Chongqing Medical University. All methods were performed in accordance with the relevant guidelines and regulations, and students and their parents had obtained informed consent prior to the investigation.

**References**

1. Cyranoski D. China tackles surge in mental illness. Nature. 2010;468(7321):145. doi: 10.1038/468145a.
2. Wang C, Zhang P, Zhang N. Adolescent mental health in China requires more attention. Lancet Public Health. 2020;5(12):e637. doi: 10.1016/S2468-2667(20)30094-3.
3. Psychiatric institutions in China. Lancet. 2010;376(9734):2. doi: 10.1016/S0140-6736(10)61039-2.
4. Zhao YX, Zheng Y. Recent progress in epidemiological studies of child mental disorders. Chinese J Psychiatry. 2014;47(3):186–9. doi:10.3760/cma.j.issn. 1006-7884.2014.03.022.
5. Xiao Y, Wang Y, Chang W, Chen Y, Yu Z, Risch HA. Factors associated with psychological resilience in left-behind children in southwest China. Asian J Psychiatr. 2019; 46:1-5. doi: 10.1016/j.ajp.2019.09. 014.
6. J Harvey, PH Delfabbro. Psychological resilience in disadvantaged youth: A critical overview. Aust Psychol. 2004,39(1). doi:10.1080/00050060410001660281.
7. Haggerty RJ, Garmezy N, Sherrod LR, Rutter M. Stress, risk, and resilience in children and adolescents: Processes, mechanisms, and interventions. Cambridge University Press, 1996.
8. Singh R, Mahato S, Singh B, Bhushal S, Fomani FK. Psychometric properties of Adolescent Resilience Questionnaire among Nepalese adolescents in Lalitpur. Asian J Psychiatr. 2019; 45:13-17. doi: 10.1016/j.ajp.2019.08.002.
9. Epstein RM, Krasner MS. Physician resilience: what it means, why it matters, and how to promote it. Acad Med. 2013;88(3):301-3. doi: 10.1097/ACM.0b013e318280cff0.
10. Mandleco BL, Peery JC. An organizational framework for conceptualizing resilience in children. J Child Adolesc Psychiatr Nurs. 2000;13(3):99-111. doi: 10.1111/j.1744-6171.2000.tb00086.x.
11. Dray J, Bowman J, Freund M, Campbell E, Wolfenden L, Hodder RK, et al. Improving adolescent mental health and resilience through a resilience-based intervention in schools: study protocol for a randomised controlled trial. Trials. 2014;15:289. doi: 10.1186/1745-6215-15-289.
12. Fenwick-Smith A, Dahlberg EE, Thompson SC. Systematic review of resilience-enhancing, universal, primary school-based mental health promotion programs. BMC Psychol. 2018;6(1):30. doi: 10.1186/s40359-018-0242-3.
13. Hodgson, R. Effective mental health promotion: a literature review. Health Educ J. 1996; 55(1):55-74. doi:10.1177/001789699605500106.
14. Akça ÖF, Ağaç Vural T, Türkoğlu S, Kılıç EZ. Anxiety sensitivity: changes with puberty and cardiovascular variables. Pediatr Int. 2015;57(1):49-54. doi: 10.1111/ped.12443. PMID: 25040018.
15. Mendle J, Harden KP, Brooks-Gunn J, Graber JA. Development's tortoise and hare: pubertal timing, pubertal tempo, and depressive symptoms in boys and girls. Dev Psychol. 2010;46(5):1341-53. doi: 10.1037/a0020205.
16. Alloy LB, Hamilton JL, Hamlat EJ, Abramson LY. Pubertal Development, Emotion Regulatory Styles, and the Emergence of Sex Differences in Internalizing Disorders and Symptoms in Adolescence. Clin Psychol Sci. 2016;4(5):867-881. doi: 10.1177/2167702616643008.
17. Deardorff J, Hayward C, Wilson KA, Bryson S, Hammer LD, Agras S. Puberty and gender interact to predict social anxiety symptoms in early adolescence. J Adolesc Health. 2007;41(1):102-4. doi: 10.1016/j.jadohealth.2007.02.013.
18. Angold A, Costello EJ, Worthman CM. Puberty and depression: the roles of age, pubertal status and pubertal timing. Psychol Med. 1998;28(1):51-61. doi: 10.1017/s003329179700593x.
19. Zhang L, Zhang D, Sun Y. Adverse Childhood Experiences and Early Pubertal Timing Among Girls: A Meta-Analysis. Int J Environ Res Public Health. 2019;16(16):2887. doi: 10.3390/ijerph16162887.
20. Sumia M, Lindberg N, Työläjärvi M, Kaltiala-Heino R. Early pubertal timing is common among adolescent girl-to-boy sex reassignment applicants. Eur J Contracept Reprod Health Care. 2016;21(6):483-485. doi: 10.1080/13625187.2016.1238893.
21. Peters AT, Burkhouse KL, Kujawa A, Afshar K, Fitzgerald KD, Monk CS, et al. Impact of pubertal timing and depression on error-related brain activity in anxious youth. Dev Psychobiol. 2019;61(1):69-80. doi: 10.1002/dev.21763.
22. Deardorff J, Ekwaru JP, Kushi LH, Ellis BJ, Greenspan LC, Mirabedi A, et al. Father absence, body mass index, and pubertal timing in girls: differential effects by family income and ethnicity. J Adolesc Health. 2011;48(5):441-7. doi: 10.1016/j.jadohealth.2010.07.032.
23. Galvao TF, Silva MT, Zimmermann IR, Souza KM, Martins SS, Pereira MG. Pubertal timing in girls and depression: a systematic review. J Affect Disord. 2014; 155:13-9. doi: 10.1016/j.jad.2013.10.034. PMID:
24. Jane Mendle. Why Puberty Matters for Psychopathology. Child Dev Perspect. 2014;8(4). doi:10.1111/cdep.12092.
25. National Health Commission of the People’s Republic of China. Healthy China Action (2019–2030). July 15, 2019. http://www.nhc.gov.cn/guihuaxxs/s3585u/201907/e9275fb95d5b4295be8308415d4cd1b2.shtml (accessed July 9, 2019; in Chinese).
26. Fenwick-Smith A, Dahlberg EE, Thompson SC. Systematic review of resilience-enhancing, universal, primary school-based mental health promotion programs. BMC Psychol. 2018;6(1):30. doi: 10.1186/s40359-018-0242-3.
27. Dray J, Bowman J, Campbell E, Freund M, Wolfenden L, Hodder RK, et al. Systematic Review of Universal Resilience-Focused Interventions Targeting Child and Adolescent Mental Health in the School Setting. J Am Acad Child Adolesc Psychiatry. 2017;56(10):813-824. doi: 10.1016/j.jaac.2017.07.780.
28. Feiss R, Dolinger SB, Merritt M, Reiche E, Martin K, Yanes JA, et al. A Systematic Review and Meta-Analysis of School-Based Stress, Anxiety, and Depression Prevention Programs for Adolescents. J Youth Adolesc. 2019;48(9):1668-1685. doi: 10.1007/s10964-019-01085-0.
29. Orton E, Whitehead J, Mhizha-Murira J, Clarkson M, Watson MC, Mulvaney CA, et al. School-based education programmes for the prevention of unintentional injuries in children and young people. Cochrane Database Syst Rev. 2016;12(12):CD010246. doi: 10.1002/14651858.CD010246.pub2.
30. Foxcroft DR, Tsertsvadze A. Universal school-based prevention programs for alcohol misuse in young people. Cochrane Database Syst Rev. 2011;(5):CD009113. doi: 10.1002/14651858.CD009113.
31. Thomas RE, McLellan J, Perera R. School-based programmes for preventing smoking. Cochrane Database Syst Rev. 2013;2013(4):CD001293. doi: 10.1002/14651858.CD001293.pub3.
32. Shackleton N, Jamal F, Viner RM, Dickson K, Patton G, Bonell C. School-Based Interventions Going Beyond Health Education to Promote Adolescent Health: Systematic Review of Reviews. J Adolesc Health. 2016;58(4):382-396. doi: 10.1016/j.jadohealth.2015.12.017.
33. Dorn LD, Hostinar CE, Susman EJ, Pervanidou P. Conceptualizing Puberty as a Window of Opportunity for Impacting Health and Well-Being Across the Life Span. J Res Adolesc. 2019;29(1):155-176. doi: 10.1111/jora.12431.
34. Diao H, Pu Y, Yang L, Li T, Jin F, Wang H. The impacts of peer education based on adolescent health education on the quality of life in adolescents: a randomized controlled trial. Qual Life Res. 2020;29(1):153-161. doi: 10.1007/s11136-019-02309-3. PMID: 31562569.
35. Khan NA, Nasti C, Evans EM, Chapman-Novakofski K. Peer education, exercising, and eating right (PEER): training of peers in an undergraduate faculty teaching partnership. J Nutr Educ Behav. 2009;41(1):68-70. doi: 10.1016/j.jneb.2008.03.116.
36. Evcili F, Golbasi Z. The effect of peer education model on sexual myths of Turkish university students: An interventional study. Perspect Psychiatr Care. 2019;55(2):239-248. doi: 10.1111/ppc.12344.
37. Parkinson, M. The effect of peer assisted learning support (PALS) on performance in mathematics and chemistry. Innov in Educ & Teach Inl. 2009;46(4):381-392. doi:10.1080/14703290903301784.
38. Puffer S, Torgerson DJ, Watson J. Cluster randomized controlled trials. J Eval Clin Pract. 2005;11(5):479-83. doi: 10.1111/j.1365-2753.2005.00568.x.
39. Handlos LN, Chakraborty H, Sen PK. Evaluation of cluster-randomized trials on maternal and child health research in developing countries. Trop Med Int Health. 2009;14(8):947-56. doi: 10.1111/j.1365-3156.2009.02313.x.
40. Wan X, Li Z, Liu J. Sample size estimation in clinical studies: (1) clinical trials (in Chinese). J Tradit Chin Med. 2007; 48:504-507. doi:10.13288/j.11-2166/r.2007.06.
41. Dong F, Li C, Peng X, Qin H. Significance, calculation methods and precautions of sample content calculation in clinical research (in Chinese). Chin J Stroke. 2009;4:854-859. doi: CNKI: SUN: ZUZH.0.2009-10-023.
42. Campbell MK, Piaggio G, Elbourne DR, Altman DG; CONSORT Group. Consort 2010 statement: extension to cluster randomised trials. BMJ. 2012;345:e5661. doi: 10.1136/bmj.e5661.
43. Eldridge SM, Ashby D, Kerry S. Sample size for cluster randomized trials: effect of coefficient of variation of cluster size and analysis method. Int J Epidemiol. 2006;35(5):1292-300. doi: 10.1093/ije/dyl129.
44. Henderson, N, Milstein, MM. Resiliency in Schools: Making It Happen for Students and Educators. Corwin Press, Inc. A Sage Publications Company, 2455 Teller Road, Thousand Oaks, CA 91,320,1996.
45. Hu Y, Gan Y. Compilation and validity verification of Resilience Scale for Chinese Adolescents (in Chinese). Acta Psychologica Sinica. 2008;(08):902-912. doi: CNKI: SUN: XLXB.0.2008-08-005.
46. Henderson, N, Milstein, MM. Resiliency in Schools: Making It Happen for Students and Educators. Corwin Press, Inc. A Sage Publications Company, 2455 Teller Road, Thousand Oaks, CA 91,320,1996.
47. Hu Y, Gan Y. Compilation and validity verification of Resilience Scale for Chinese Adolescents (in Chinese). Acta Psychologica Sinica. 2008;(08):902-912. doi: CNKI: SUN: XLXB.0.2008-08-005.

**Research protocol: part 2**

### **Budget**

The total budget of this study is RMB 60,000, and the details are shown in Table 2.

### Table 2 The budget of the research funds

| Category | Amount (¥) | Illustrate |
| --- | --- | --- |
| Direct [expenses](D:/LenovoSoftstore/Install/wangyiyoudaocidian/8.9.6.0/resultui/html/index.html#/javascript:;) | 4 0000 |  |
| Books and materials | 3 000 | Peer education training materials, appliances; Student books, promotional materials |
| Data collection | 3 000 | Baseline and follow-up surveys |
| Conference/travel/international cooperation and exchange | 20 000 | Project kick-off meeting, peer education and training session, travel expenses for baseline and survey staff |
| Expert consultation | 3 000 | Expert consultation on peer education issues |
| [Service](D:/LenovoSoftstore/Install/wangyiyoudaocidian/8.9.6.0/resultui/html/index.html#/javascript:;) [charge](D:/LenovoSoftstore/Install/wangyiyoudaocidian/8.9.6.0/resultui/html/index.html#/javascript:;) | 9 000 | Data entry for baseline and follow-up surveys |
| Printing expenses | 2 000 | Survey materials, meeting documents, intervention materials, supervision manuals and other materials |
| Indirect [expenses](D:/LenovoSoftstore/Install/wangyiyoudaocidian/8.9.6.0/resultui/html/index.html#/javascript:;) | 20 000 |  |

### **Other support for the project**

The study was supported by Social and Humanities Sciences Research Planning Fund Project from Ministry of Education (17YJA840015) and Research Special Fund for Public Welfare Industry of Health (No.201202010).
